# Combination of a triple alpha-globin gene with beta-thalassemia in a gypsy family: importance of the genetic testing in the diagnosis and search for a donor for bone marrow transplantation for one of their children

**DOI:** 10.1186/s13104-016-2027-1

**Published:** 2016-04-14

**Authors:** Flor Yus Cebrian, María del Valle Recasens Flores, Silvia Izquierdo Álvarez, Ingrid Parra Salinas, Carmen Rodriguez-Vigil Iturrate

**Affiliations:** Department of Hematology, Hospital Universitario Miguel Servet, C/Padre Arrupe, s/n, planta 4ª, 50009 Saragossa, Spain; Genetics Unit, Department of Clinical Biochemistry, Hospital Universitario Miguel Servet, Saragossa, Spain; Department of Pediatrics, Hospital Universitario Miguel Servet, Saragossa, Spain

**Keywords:** Splenomegaly, Microcytic anemia, Beta thalassemia major, Thalassemia minor, Iron overload, Alpha-globin gene triplication

## Abstract

**Background:**

The simultaneous presence of a heterozygous β-thalassemia with α-gene triplication may cause anything from a thalassemia trait to thalassemia intermedia of mild to moderate severity.

**Case presentation:**

An 8-month-old ethnic Gypsy male infant with failure to thrive from birth, mild jaundice and splenomegaly. Clinical signs were compatible with severe microcytic anemia requiring bi-monthly blood transfusions. The β-thalassemia gene analysis found homozygous mutation IVS-I-110 (G>A) (c.93-21G>A) in intron 1 of the hemoglobin beta globin gene and a non-pathogenic sequence variant (single nucleotide polimorfism (SNP) Rs1609812). In addition, the patient had α gene triplication (ααα^anti 3.7^/αα) caused by double heterozygosity for a 3.7 kb fragment that contained only the hemoglobin alpha globin gene-2 gene. This finding led to screening and follow up in first-degree relatives, twin brothers and a sister and parents to provide them with appropriate genetic counseling. Nowadays, new horizons could open a new therapeutic management until definitive cure of these diseases through gene therapy or mutation-specific genome editing.

**Conclusions:**

Genetic testing can provide an early diagnosis and facilitates the search for a suitable donor for transplantation.

## Background

Thalassemia syndromes are a group of hemoglobinopathies characterized by gene defects that disrupt hemoglobin synthesis. β-thalassemia results from insufficient (β^+^) or absent (β^0^) synthesis of normal hemoglobin chains. The molecular basis involves mutations in β-globin genes (mostly point mutations). Most patients are from Mediterranean countries, Southeastern Europe, Arab and Asian countries [1]. Hematological changes do not show up before patients are 3–6 months old [1, 2].

The imbalance between the α- and β-globin chains is central to the pathophysiology of β-thalassemia. In homozygous β-thalassemia, the excess of α-globin chains precipitates on the membranes of the red cell precursors of the bone marrow causing hemolysis, ineffective erythropoiesis and anemia [3, 4].

There are still some unknown aspects in the relationship between genotype and phenotype in thalassemias, such as the simultaneous presence of heterozygous β-thalassemia with triple α-gene. This combination may cause anything from a thalassemia trait to thalassemia intermedia of mild to moderate severity, as in the case presented here.

## Case presentation

An 8-month-old male infant, Portuguese of gypsy ethnic origin, who presented with marked failure to thrive from birth, mild jaundice of the skin and splenomegaly (greatest longitudinal diameter was 87 mm at 7 months). An initial differential diagnosis was made to distinguish between hemoglobinopathies and other types of anemia (deficiency anemia, hemolytic anemia, erythroblastopenia). The clinical signs were compatible with severe microcytic anemia, requiring bi-monthly blood transfusions from the time of diagnosis. Admission to hospital was needed on several occasions due to infectious episodes (gastroenteritis due to *Salmonella*, *Parvovirus B19* seroconversion) and for the implantation of an intravenous (IV) reservoir because of the transfusion requirements.

Blood parameters were analyzed with the COULTER LH 780 Series (Beckman), a hematology analyzer used to count and determine the size of blood cells. Hemoglobin analysis and the quantification of hemoglobin (Hb) A2 and Hb F were performed using Minicap Flex Piercing (Sebia) capillary electrophoresis.

The analysis of deoxyribonucleic acid (DNA), taken from peripheral blood with ethylenediaminetetraacetic acid (EDTA), was performed using proteolytic digestion followed by purification and ethanol precipitation. Next, spectrophotometry was applied to quantify the obtained DNA. All coding exons and adjacent intronic regions of the HBB gene (hemoglobin beta globin gene) as well as the CACC-box (cytosine-adenine-cytosine-cytosine-box) in the proximal promoter region and 5′UTR and 3′UTR regions were amplified using PCR (polymerase chain reaction).

In the testing for α-thalassemia, hybridization of the DNA that was obtained was performed using specific probes that are complementary to chromosomal region 16 p13.3, which contains the HBA (hemoglobin alpha globin) gene cluster. Subsequently PCR amplification of the genes located in the HBA gene cluster was performed using the MLPA (multiplex ligation-dependent probe amplification) technique.

Lastly, sequencing of both strands of the amplified fragments was performed and the sequences were visualized by capillary electrophoresis using the Applied Biosystems© 3500 Dx Genetic Analyzer.

Labgenetics (Laboratorio de Genética Clínica S.L.) performed the genetic testing to diagnose α- and β-thalassemia.

**Fig. 1:**
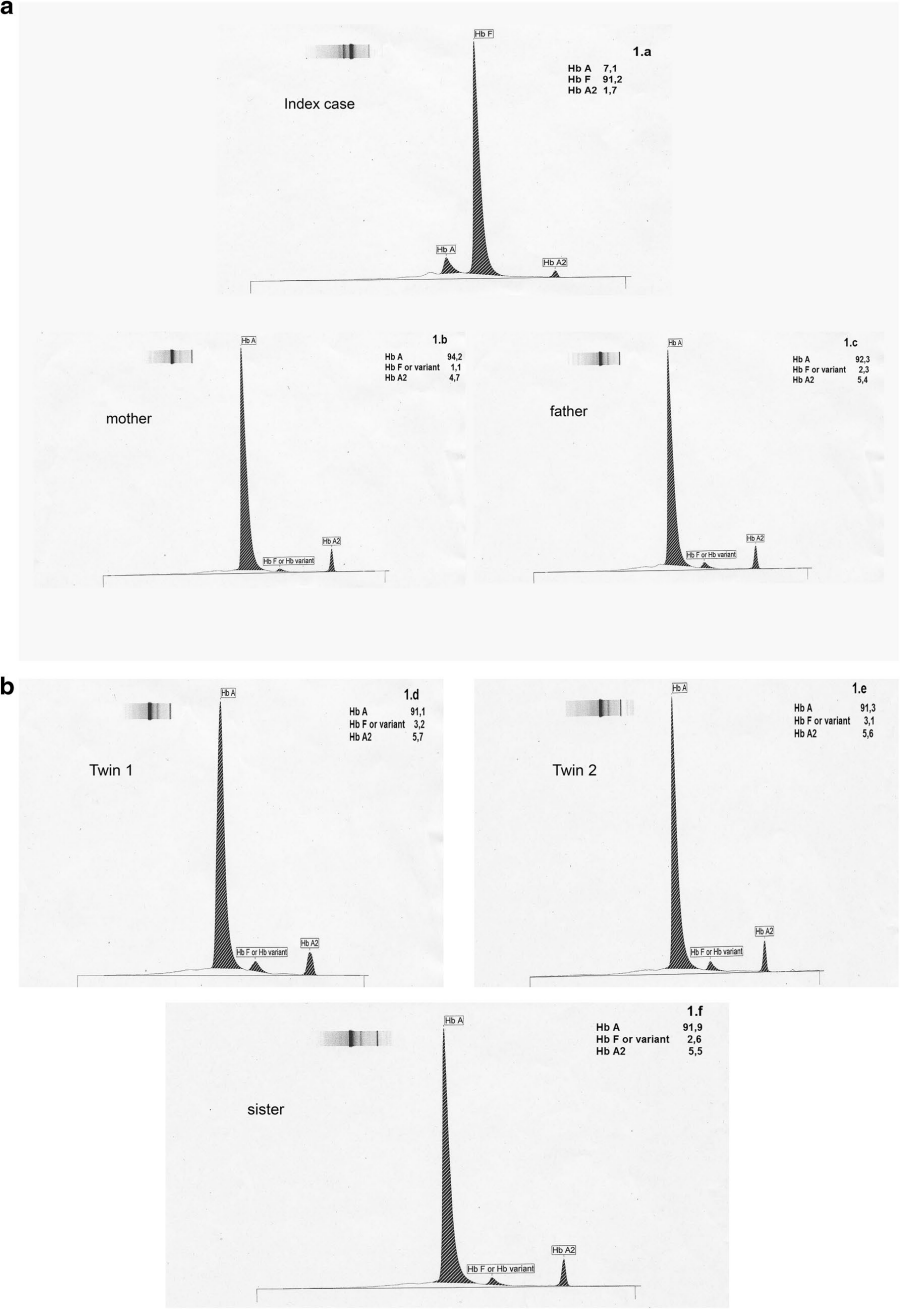
HPLC images of (**a**) index case, mother and father, (**b**) sister and brothers (twins)

The patient’s laboratory findings when he was 8 months old were as follows: microcytic anemia without other laboratory abnormalities, with Hb 6.2 g/dL, hematocrit (Hct) 20 %, mean corpuscular volume (MCV) 74.40 fL, mean corpuscular hemoglobin (MCH) 22.90 pg, without iron deficiency. HbA2 1.7 %, HbF 90.0 % (see Fig. 1). Biochemical values revealed: lactate dehydrogenase (LDH) 49 mg/dL, total bilirubin (TBIL) 1.40 mg/dL, direct bilirubin (DBIL) 0.31 mg/dL and reticulocytosis (4.14 %) (see Table 1).

**Table 1:** Analytical results of the index case and family

	Index case (8 months old)	Twin 1	Twin 2	Sister	Mother	Father	Rev. values
Gender	♂	♂	♂	♀	♀	♂	–
α genotype	ααα^anti 3.7^/αα	ααα^anti3.7^/ααα^anti3.7^	ααα^anti3.7^/ααα^anti3.7^	Ααα^anti3.7^/-	ααα^anti3.7^/αα	ααα^anti3.7^/ααα^anti3.7^	–
β genotype	β^+^/β^+^	β^+^/N (normal)	β^+^/N (normal)	–	β^+^/N (normal)	β^+^/N (normal)	–
HbA2	1.7	5.6	5.70	5.5	4.7	5.4	–
HbF	90.0	3.10	3.20	2.60	1.1	2.3	–
Hb (gr/dL)	6.2	10.8	9.3	10.0	8.5	11.2	13.2–18.0
Hct (%)	20.0	34.9	29.8	31.9	27.5	35.9	39.0–51.0
Red blood cells (×10^9^/L)	2.68	5.92	5.11	5.47	4.00	5.89	4.32–5.66
MCV (fL)	74.40	58.80	58.20	58.40	68.80	60.90	80.0–98.0
MCH (pg)	22.90	18.20	18.20	18.30	21.30	19	27.30–32.60
Reticulocytes (%)	4.14	1.57	2.30	2.70	3.65	–	0.80–2.50
Leukocytes (×10^9^/L)	16.0	10.9	12.1	6.40	8.6	8.4	3.7–9.5
Platelets (×10^9^/L)	150	351	398	238	263	230	125–450
Ferritin (ng/mL)	99.9	64.2	95.8	147.7	16.2	743.4	12–300
LDH (mg/dL)	449	350	388	369	233	–	180–430
Total bilirubin (mg/dL)	1.40	0.37	1.01	1.35	0.60	–	0.30–1.20
Direct bilirubin (mg/dL)	0.31	0.09	–	0.26	–	–	0.0–0.20
Clinical signs and symptoms	Severe anemia, failure to thrive, splenomegaly	Mild/moderate anemia	Mild/moderate anemia	Mild anemia	Moderate anemia, menorrhagia	Mild anemia	–

The β-thalassemia gene analysis found homozygous mutation IVS-I-110 (G>A), (c.93-21G>A), in intron 1 of the HBB gene and a non-pathogenic sequence variant [SNP (Single nucleotide polimorfism) Rs1609812], with heterozygous mutation IVS-II-666 (T>C) in intron 2 of the HBB gene.

Mutation IVS-I-110 (G>A), (c.93-21G>A), in intron 1 of the HBB gene can cause protein splicing defects, leading to aberrant mRNA (ribonucleic acid) and thus result in the synthesis of a non-functional protein. The presence of this homozygous mutation implies a diagnosis of β-thalassemia major. The presence of α gene triplication in heterozygosis has no effect on the synthesis of α globin chains in hemoglobin and subjects are usually clinically asymptomatic. However, the combination of a triplicated α-globin gene together with heterozygous β-thalassemia may produce a thalassemia intermedia phenotype, as it increases the imbalance between the α- and β-globin chains [4, 5].

At the age of 2 years the child required monthly transfusions and had marked failure to thrive (weight and height below P3, head circumference P10), splenomegaly and required chelation therapy with Deferasirox EXJADE^®^ (deferasirox) (10 mg/kg/day) as maintained iron overload was found. The study of the whole family is normally done routinely.

**Fig. 2:**
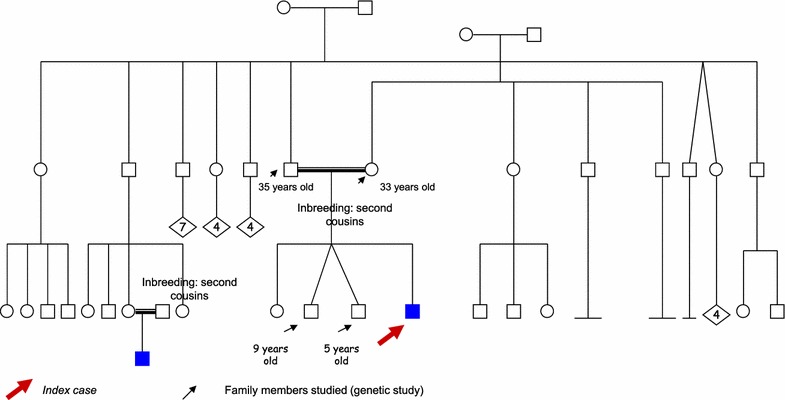
Pedigree with the results for family members

The subject had two twin brothers (fraternal twins) aged 5 years, who were clinically asymptomatic at the time the analysis of the index case was performed (see Fig. 2, the family gave its consent to carrying out the family tree as part of routine study). Laboratory tests revealed they had mild microcytic anemia (Hb 9.3–10.8 g/dL, Hct 29.8–34.9 %, MCV 58.80 fL, MCH 18.20–22.90 pg) (see Table 1; Fig. 1b). Gene testing in both revealed that they had heterozygous mutation IVS-I-110 (G>A), (c.93-21G>A), in intron 1 of the HBB gene and were homozygous for α-gene triplication (ααα^anti 3.7^/ααα^anti 3.7^).

The heterozygous mutation IVS-I-110 (G>A), (c.93-21G>A), in intron 1 of the HBB gene can result in the synthesis of a non-functional aberrant protein. This nucleotide change has been described as a mutation associated with the development of β-thalassemia. The homozygous α-gene triplication (ααα^anti 3.7^/ααα^anti 3.7^) causes an increase in the synthesis of the α-globin chain of hemoglobin, leading to a slight increase in the level of hemoglobin in the blood and resulting in an imbalance in α- and β-chain hemoglobin synthesis. The combination of a homozygous or heterozygous triplicated α-globin gene together with heterozygous β-thalassemia would produce a thalassemia intermedia phenotype, as the imbalance between the α- and β-globin chains would increase [4, 5].

The subject also had a 9-year-old sister (see Fig. 2) with thalassemia minor who was clinically asymptomatic, she had a microcytic anemia without other laboratory abnormalities (Hb 10.0 g/dL, Hct 31.9 %, MCV 58.0 fL, MCH 18.30 pg, HbA2 5.5 %, HbF 2.6 %, HbA 91.9) (see Table 1; Fig. 1a), and she had at least a triplicated alpha globin gene in heterozygosis. The mother was diagnosed with thalassemia minor and she also presented with severe iron deficiency because of menorrhagia that often needed to be treated with intravenous iron (Hb 8.5 g/dL, Hct 27.5, MCV 68.8 fL, MCH 21.30 pg, HbA2 4.9 % and HbF 1.4 %) (see Table 1; Fig. 1a, b). The genetic study revealed heterozygosity for the IVS-I-110 (G>A) (c.93-21G>A) mutation in intron 1 of the HBB gene and heterozygosity for alpha-gene triplication (ααα^3.7^/αα).

The father was clinically asymptomatic and laboratory tests showed he had mild microcytic anemia (Hb 11.2 g/dL, Hct 35.9 %, MCV 60.90 fL, MCH 19 pg) (see Table 1; Fig. 1a). He had heterozygosity for the IVS-I-110 (G>A) (c.93-21G>A) mutation in intron 1 of the HBB gene and was homozygous for alpha-gene triplication (ααα3.7/ααα3.7). Blood samples were taken from parents and sibilings as a part of routine care of patient. Parents signed informed consent for molecular genetic studies for themselves and their children.

## Discussion

The index case presented with failure to thrive (he was below the percentile for his sex and age) and was diagnosed with severe microcytic anemia. His transfusion requirements have increased and at present are required on a monthly basis. At the age two he started chelation therapy with deferasirox (10 mg/kg/day) due to the progressive increase in serum ferritin levels and other parameters indicative of iron overload. Chelation therapy for the treatment of iron overload in patients with β-thalassemia is indicated from the age of 2 years, following lab tests to rule out changes in renal and liver function [6]. This treatment is well tolerated by the patient, and can be given orally without need of discontinuation of other treatment; in subsequent follow up showed no effect on creatinine and transaminase levels. In adition, this drug dose has been adjusted to the subject’s weight, with good response to ferritin levels, but it has not been made liver MRI (magnetic resonance imaging).

Given the patient’s condition and since β-thalassemia major was confirmed by genetic testing, we decided to perform an HLA study in family members in order to undertake allogeneic hematopoietic stem cell transplantation from a related donor. The index case was found to be an identical HLA match to his twin brothers, but screening for hemoglobinopathies and genetic testing found that the brothers had heterozygous β-thalassemia together with α-gene triplication (see Fig. 1b; Table 1). For this reason it was decided that the brothers would not be suitable donors for a hematopoietic stem cell transplantation as this combination of inherited factors involves a broad clinical spectrum that is highly unpredictable, so at this time we cannot be certain about the clinical implications of the donors also being affected by the condition, despite being asymptomatic at present. The search for an unrelated donor was made in the Bone Marrow Donors Registry (*Registro de donantes de médula ósea*, REDMO) and a suitable candidate was found.

Although there have been numerous advances in the molecular characterization of thalassemia, at present certain aspects of the relationship between genotype and phenotype remain unknown; one of these is the simultaneous presence of a heterozygous β-thalassemia with α-gene triplication. This interaction may cause anything from a thalassemia trait to thalassemia intermedia of mild to moderate severity [3, 4, 7]. However, the index case has the homozygous mutation IVS-I-110 (G>A), (c.93-21G>A) and the presence of α gene triplication in heterozygosis, this genetic association clinically manifested as severe anemia. And his brothers presented the heterozygous mutation IVS-I-110 (G>A) and α gene triplication, and they have an anemia less severe. Perhaps we should consider the role of the mutation IVS-I-110 (G>A) and its association with α gene triplication in terms of clinical character. The insufficient growth may have many causes and a transfusion per month may be normal in a major patient [8].

β-thalassemia is a benign congenital blood disorder, but patients with β thalassemia major need regular blood transfusions throughout their whole lives and this leads to iron overload that causes progressive organ dysfunction. With the currently available treatments, over 80 % of subjects have a life expectancy of more than 35 years [9, 10]. Still, the only curative treatment for the disease is hematopoietic stem cell transplantation. Subjects who have an HLA-identical healthy sibling are candidates for transplantation, although if this is not the case, at present unrelated donor transplant outcomes are very promising, expanding the possibilities for these patients [10, 11].

These genetic and clinical findings have important implications for prenatal screening and genetic counseling programs. Families in which heterozygosity for α-gene triplication and β-thalassemia coexists in both parents may have a child with thalassemia intermedia or major. In our case, it is essential to provide adequate genetic counseling to the family because of their family history for the disease, which in some cases has even required bone marrow transplantation (see Fig. 2). It is also necessary to provide them with an explanation of the clinical implications of the disease and, in case of pregnancy, to offer them the possibility of having prenatal genetic testing performed on the fetus [4].

Mettananda et al. reported the reduction of α-globin expression would ameliorate the clinical severity of patients with β-thalassemia. It seems that therapeutic regulation of α-globin expression is feasible by RNA interference, epigenetic drug targeting, or genome editing. Therefore, exploring new horizons could open a new therapeutic management until definitive cure of these diseases through gene therapy or mutation-specific genome editing [12].

## Conclusions

The simultaneous presence of a heterozygous β-thalassemia with α-gene triplication may cause differences in the genotype-phenotype correlation this is why genetic testing can provide an early diagnosis and facilitates the search for a suitable donor for transplantation when needed (for example, of hematopoietic stem cells).
